# An Eccentrically Biased Rehabilitation Program Early after TKA Surgery

**DOI:** 10.1155/2011/353149

**Published:** 2011-04-07

**Authors:** Robin L. Marcus, Yuri Yoshida, Whitney Meier, Christopher Peters, Paul C. LaStayo

**Affiliations:** ^1^Department of Physical Therapy, University of Utah, Salt Lake City, UT 84108, USA; ^2^Department of Orthopaedics, University of Utah, Salt Lake City, UT 84108, USA

## Abstract

Rehabilitation services are less-studied aspects of the management following total knee arthroplasty (TKA) despite long-term suboptimal physical functioning and chronic deficits in muscle function. This paper describes the preliminary findings of a six-week (12 session) eccentrically-biased rehabilitation program targeted at deficits in physical function and muscle function, initiated one month following surgery. A quasiexperimental, one group, pretest-posttest study with thirteen individuals (6 female, 7 male; mean age 57 ± 7 years) examined the effectiveness of an eccentrically-biased rehabilitation program. The program resulted in improvements in the primary physical function endpoints (SF-36 physical component summary and the six-minute walk test) with increases of 59% and 47%, respectively. Muscle function endpoints (knee extension strength and power) also increased 107% and 93%, respectively. Eccentrically-biased exercise used as an addition to rehabilitation may help amplify and accelerate physical function following TKA surgery.

## 1. Introduction

In the US, over 500,000 total knee arthroplasty (TKA) surgeries are performed each year for severe knee osteoarthritis and that number is expected to increase sevenfold over the next two decades [1]. Most TKA recipients experience a successful reduction of their knee pain and an improvement in knee function [2, 3]. Unfortunately, not all TKA recipients experience substantial improvements in their levels of pain, functional status, nor overall health-related quality of life. That is, more than one-third of TKA recipients have suboptimal physical function [4–7]. Further, almost all TKA recipients report levels of physical function that are below age-matched normative levels [8] and their performance-based physical function scores rarely reach levels of control subjects [9]. Walking speeds are up to one-third slower [10], the time to negotiate stairs is two-times slower [11] and the distance walked in six minutes is 20% shorter [12] than control subjects. 

Impairments in muscle function (e.g., strength) parallel these physical function deficiencies. Following TKA surgery, muscle weakness is ubiquitous and directly associated with impaired physical functioning [9, 13–15]. A recent review [16] exposes deficits (>20%) in quadriceps muscle strength and power six months to 13 years after TKA surgery. While muscle strength does increase steadily in the first three to six months after surgery, these improvements taper off thereafter [13]. That is, strength improves 10–20% but rarely ever reaches that of age-matched individuals with nonarthritic native knees [17, 18] or that of the contralateral nonoperative knee extensor muscles [9, 11, 17, 19, 20]. 

Many institutions, centers, and clinics provide outpatient therapy to patients following TKA surgery. Despite this, a best-practice outpatient rehabilitation approach has not been published. This void in the peer-reviewed literature, highlighted by an NIH-sponsored expert panel [21] as one of the most understudied aspects following TKA, underscores the need for additional evidence. A recent meta-analysis on the topic [22] substantiated the short-term benefit of TKA rehabilitation; however, the limited number of muscle-focused TKA rehabilitation trials exposes the fact that higher intensity resistance exercises are not regularly employed in this population. In fact, only one previous RCT compared progressive leg-strengthening exercises coupled with electrical stimulation of the quadriceps (17 sessions) to a standard of care group (23 sessions) [15]. While the six weeks of resistance exercise demonstrated superior strength and mobility improvements over the standard of care at one year, there were no group differential effects on the self-report of physical function as depicted by the Short-Form 36 Healthy Survey Physical Component Summary (SF-36_PCS_) [15]. 

It is our contention that high muscle force producing resistance exercise can amplify muscle conditioning and optimize the level of physical function to that of healthy normative groups. Our laboratory has specifically studied the effects of resistance exercise via negative, eccentrically-induced work on muscle and mobility function in older adults with a variety of comorbid conditions. Significant improvements following eccentric exercise have been noted in muscle strength and size and in the hallmark activities underlying physical function (e.g., the ability to walk and negotiate stairs) [23–27]. Importantly, eccentrically-biased rehabilitation initiated one to four years after TKA has demonstrated muscle growth and mobility responses that exceed that following traditional rehabilitation [23], but an eccentrically-biased postoperative approach has not been tested early after TKA surgery.

In this paper, we present a descriptive report of the use of an eccentrically-biased postoperative rehabilitation intervention targeted at deficits in physical function and muscle function following TKA surgery. This preliminary study examines the efficacy of a six week (12 sessions) eccentrically-biased rehabilitation program initiated one month following TKA surgery.

## 2. Materials and Methods

This study was approved by the Institutional Review Board at the University of Utah and was in accordance with the Declaration of Helsinki. All participants provided written informed consent prior to participation. A quasiexperimental, one group, pretest-posttest design was used. The same investigator (YY) collected pre and posttest measures. Potential participants were recruited sequentially from one orthopaedic surgeon's two-week postoperative TKA clinic from June 2008 through June 2009. Measurements of physical function and muscle function were assessed prior to outpatient rehabilitation (third postoperative week) and one week following the rehabilitation period (eleventh postoperative week). The six-week outpatient rehabilitation began the fourth postoperative week. 

### 2.1. Participants

**Figure 1:**
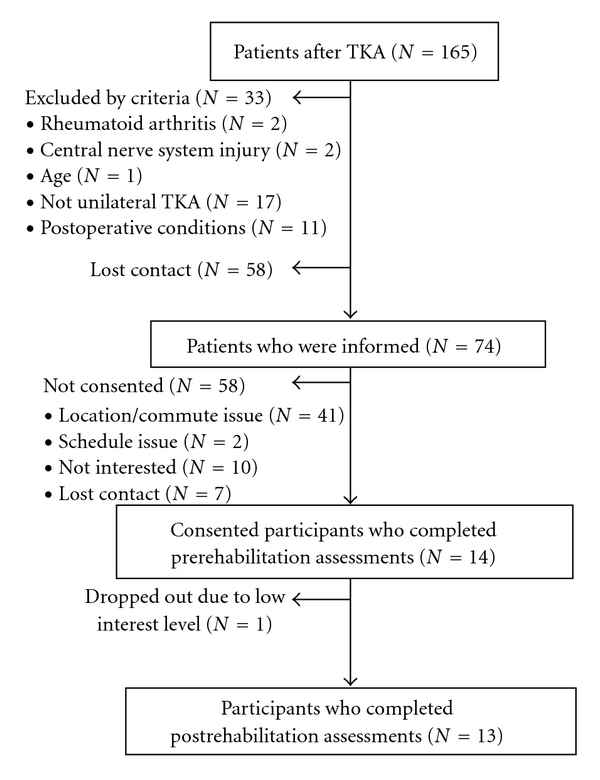
Recruitment diagram.

Thirteen individuals (6 female, 7 male) (see Table 1 and Figure 1) met the criteria for inclusion into the rehabilitation program. Participants were between the ages of 40–70 years who underwent a primary TKA for end-stage osteoarthritis and were medically cleared by their orthopaedic surgeon for rehabilitation at their second postoperative week. If individuals had a previous TKA, it must have been performed on the opposite leg at least six months prior to the current TKA. Additionally, individuals were excluded if they were not (1) discharged to home after receiving standard inpatient care, (2) able to get to the outpatient rehabilitation clinic 2x per week for 6 weeks, and (3) able to attain 90 degrees of knee flexion with a healing surgical incision without persistent wound drainage nor infection in the TKA knee at the third postoperative week. Potential participants also needed to be free of any other orthopedic issues that adversely affected their function and medically cleared to participate in rehabilitation, as determined by the referring orthopaedic surgeon.

### 2.2. TKA Surgery

All TKA procedures were performed through a minimedial parapatellar arthrotomy with minimal patella eversion [28, 29]. A conventional tricompartmental replacement was performed in all cases with the Biomet Vanguard knee system (Biomet, Warsaw, IN). After TKA, all participants were instructed by a physical therapist in weight bearing to tolerance with an assistive device and underwent inpatient care and home health physical therapy visits for the first two weeks after surgery. Inpatient care consisted of assisted walking within 24 hrs, knee range of motion exercises and preparing for return to home. Once home, the participants received one or two visits per week for progressive exercise and instruction including knee range of motion, gait training, neuromuscular electrical stimulation to augment quadriceps muscle activation, and instruction in negotiating stairs and community-related obstacles.

### 2.3. Rehabilitation Sessions

Outpatient rehabilitation was supervised by a physical therapist and performed two times per week over a six-week period. Each of the 12 rehabilitation sessions lasted 60 minutes and included a warmup, stretching, and a progressive suite of lower extremity joint range of motion exercises, resistance exercises of the lower extremities, functional task-oriented exercises, endurance exercise and a short session of cyrotherapy. All rehabilitation components were initiated at the start of rehabilitation though the intensity, frequency and duration were adjusted based on the response to treatment. Any signs of adverse knee joint responses (e.g., increased swelling or pain) resulted in a lowering of the intensity, frequency and duration of the exercises or elimination of a rehabilitation component. The resistance exercise component of each rehabilitation session utilized resistance exercise via negative, eccentrically-induced work as the major mode of lower extremity strengthening (Table 2).

### 2.4. Eccentrically-Biased Rehabilitation

**Figure 2:**
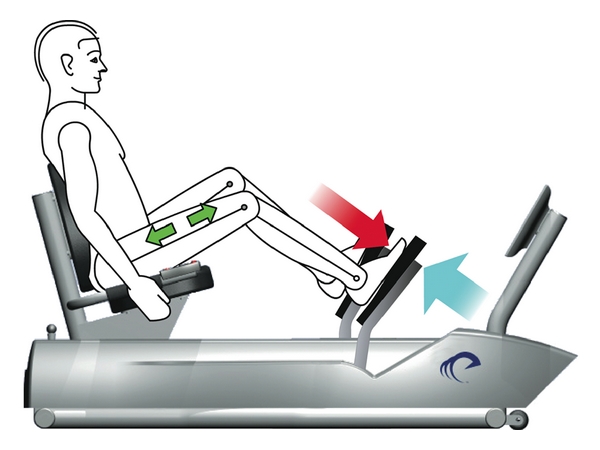
Recumbent eccentric stepper (Eccentron; BTE Technologies, Inc., Hanover, MD, USA). High muscle forces are generated on an eccentric stepper powered by a 3 hp motor that drives the pedals. As the pedals move toward the participant (blue arrow), the rider resists by applying force to the pedals (red arrow). Because the magnitude of force produced by the motor exceeds that produced by the rider, the leg extensors (green arrows) work eccentrically (lengthening), creating negative work.

Resistance exercise via negative, eccentrically-induced work (RENEW) of both the operative and nonoperative lower extremities was performed on a recumbent ergometer that appears like a normal stepper ergometer (Eccentron, BTE Technologies Inc., Hanover, MD) [23]. RENEW is quite different from a typical strengthening exercise during which the muscle must work in a concentric fashion (e.g., lifting a weight) prior to being exercised eccentrically (e.g., lowering a weight). This requisite preceding concentric action was eliminated in RENEW. While resisting the ergometer's foot pedal movement (with verbal instructions to “try to slow down the pedals”), the participant experienced eccentric muscle contractions about the knee and hip while performing negative work (see Figure 2 and description of the ergometer in [24]). The ergometer provides real-time feedback to the participants regarding the amount of resistance they are providing as it monitors the participants' resistance to the pedals and their negative work. The RENEW program was designed to progressively increase in intensity while avoiding muscle damage and has been described in detail previously [23]. Briefly, the progression of the two times per week, six-week RENEW program was determined as a function of the participants' rating of perceived exertion (RPE). A “target” workload on a computer monitor is used as feedback to guide the participant as they attempt to reach a total work level that exceeded that of the previous training session [30]. The RENEW exercise progression proceeded from a perceived exertion level of “very very light” to “somewhat hard” and from a duration of 5 minutes to 20 minutes per session (Table 3). Prior to training, the stepper seat setting was individually adjusted to each participant's leg length and safety guidelines were reviewed. RENEW was performed between 12 and 18 RPM. A visual analog scale (VAS) was used to monitor muscle and joint pain prior to each session, and heart rate and RPE were collected at the halfway point of each session.

### 2.5. Physical Function Primary Outcome (Self-Report and Performance-Based) Measurements

The *Physical Component Summary of the Short Form-36* (SF-36_PCS_) is a composite measure of physical function derived from the overall Short Form-36 [31]. The SF-36_PCS_ composite measure provides a simplified metric of physical function that accounts for all 8 subscales of the original Short Form-36 [31] and provides a normative reference score (a value of 50) representing the general US population in 1998 [32]. The SF-36_PCS_ is highly reliable (ICC = 0.90) and can readily detect changes in physical function with 5 points being the minimum clinically important difference [33]. Participants filled out the Short Form-36 using a self-administered format over a 10–15-minute period in a quite room with the research associate available to answer any questions. The SF-36_PCS_ was assessed as the primary self-report endpoint for physical function. 

The *6-Minute Walk Test* (6MW) [34, 35] is a performance-based measure of physical function (i.e., ‘‘*the ability to move around*” [36]) in adult populations, is responsive to change, and is clinically important if change scores exceed 60 m in patients after TKA [37]. The 6MW test required individuals to walk as fast and as far as they can (with or without a gait assistive device) in 6 minutes in a premeasured square course (1 lap = 89.5 meters) with verbal feedback on time elapsed and verbal encouragement [35]. The 6MW exhibits high reliability (ICC = 0.94) in patients after TKA [37]. The 6MW test was assessed as the primary performance-based endpoint for physical function. 

### 2.6. Physical Function Secondary Outcome Measurements

The *lower extremity function scale* (LEFS) is a self-report, region-specific questionnaire [38]. The LEFS is a 20-item measure of lower extremity functional status. Each item is scored on a five-point scale (0–4). Total LEFS scores can vary from 0 to 80 with higher scores being associated with greater levels of functional status. The test-retest reliability is high (ICC = 0.94) and 9 constitutes a minimal clinical important change [38]. Participants filled out the LEFS using a self-administered format over a 10–15-minute period in a quite room with the research associate available to answer any questions. 

The *Stair Climbing Test* (SCT) is one of the most demanding tasks for individuals after TKA [11]. The SCT is a reliable (ICC = 0.90) measurement of how long an individual takes to go up and down a flight of 10 stairs, and a change score of 5.5 s is considered clinically important [37]. The participants were asked to ascend and descend as quickly as they could in a safe and comfortable manner (with or without the use of a railing). The average of the three trials with the combined ascent and descent times were analyzed.

A *Gait Speed* (GS) assessment at a comfortable, self-selected speed was performed over a 10 m path and timed with a stopwatch. This test is highly reliable (ICC = 0.91) [37], and a change score of 0.1 m/s is considered clinically important [39]. The average of three trials was analyzed. 

In order to track active range of motion (ROM) *knee extension deficits* and *knee Flexion ROM *measures of flexion and extension were recorded to the nearest degree using a goniometer.

### 2.7. Muscle Function Primary Outcome Measurements

A test of *Knee Extension Maximal Voluntary Isometric Contraction* (Strength_MVIC_) torque was assessed in both the operative and nonoperative legs. Strength_MVIC_ of the quadriceps femoris muscle was evaluated at 60° of knee flexion on a dynamometer (Kin-Com 500 H, Chattecx Corp.; Harrison, TN). Participants were tested three times with a two-minute rest between trials to avoid muscle fatigue. The average maximum torque value from the three trials was analyzed. The Strength_MVIC_ techniques, which were corrected for the influence of gravity, have demonstrated high reliability (ICC = 0.99) with important change scores being 25 Nm [40]. A measure of *leg extensor maximal voluntary power output* (Power_MVPO_) is a reliable (ICC = 0.91) and important variable related to measures of physical function [41] following TKA [42, 43]. We measured Power_MVPO_ in both the operative and nonoperative legs using the Leg Extension (Nottingham) Power Rig (Queen's Medical Centre, Nottingham, UK). Following 4-5 practice trials, the participant performed 5 leg extension maximal efforts. The average maximal effort leg extension power was analyzed. The leg extension power rig is a feasible means of assessing muscle power across the lifespan [44].

### 2.8. Statistical Analysis

Descriptive statistics were calculated for demographic variables and the primary and secondary dependent measures. The RPE, work, and pain measurement results were examined graphically. The assumptions of parametric statistical tests for the primary and secondary outcomes were assessed via tests of normality and homogeneity of variance. In all cases, the assumptions were met; therefore parametric tests were performed. First, a paired *t*-test was used to test for differences between the prerehabilitation values and the postrehabilitation values for the dependent variables. The level of statistical significance for all tests for differences was set at *P* < .05. Next, to better understand the effects of the eccentrically-biased rehabilitation program, the magnitude of changes within the groups was calculated. The interval estimators of pre- to postrehabilitation changes (95% confidence intervals of the changes) as well as calculation of a within-group effect size (ES) were also analyzed.

## 3. Results

Of the total of 14 individuals who consented to participate, 13 individuals completed the full dose of eccentrically-biased rehabilitation (12 sessions over 6 weeks) and all study measures (see Figure 1). The one participant who dropped out declared that he was no longer interested in pursuing rehabilitation. There were no adverse events. 

**Figure 3:**
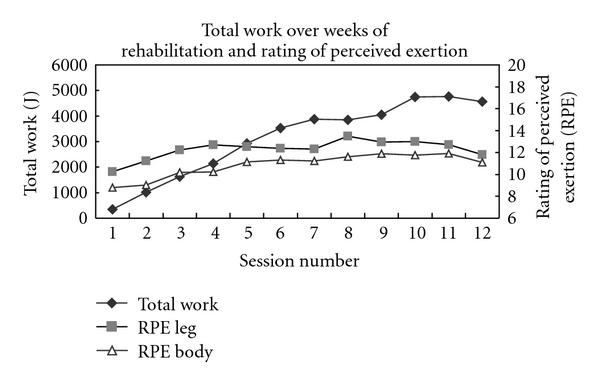
Total work of RENEW over the weeks of rehabilitation and the respective ratings of perceived exertion.

**Figure 4:**
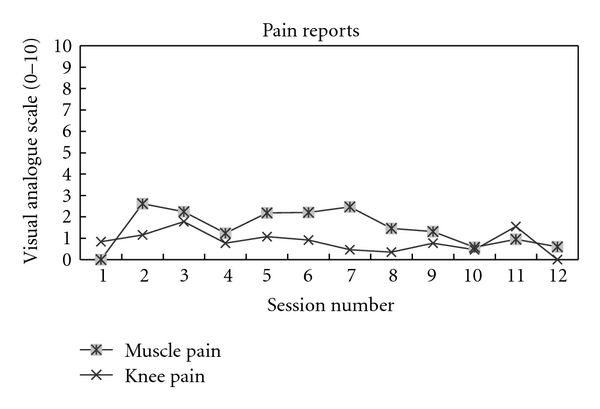
Reported level (visual analog scale) of muscle and knee pain over the weeks of rehabilitation with RENEW.

The total amount of work during RENEW increased approximately 5-fold over the course of the 6 weeks of rehabilitation despite a perceived level of exertion never exceeding “somewhat hard” (Figure 3). During this time, the mean perceived muscle and knee pain on a 0–10 VAS never exceeded a level of 3 over the first half of the rehabilitation sessions and diminished to approximately a level of 1-2 over the second half of the rehabilitation sessions (Figure 4). 

Participants significantly (*P* < .01) improved on all physical function outcomes and muscle function outcomes related to the operative leg (Table 4). The primary physical function endpoints, SF-36_PCS_ and 6MW, increased 59% and 47%, respectively. Gait assistive devices were used by 46% of the participants during the 6MW test prior to rehabilitation, but following the six weeks of exercise, none used an assistive device. The secondary physical function endpoints also improved with LEFS increasing 55% SCT improving 47%, and GS increasing 30%. During SCT, a handrail was used by every participant prior to rehabilitation, but following six weeks of rehabilitation, a handrail was used 65% of the time. Finally, the primary muscle function endpoints of the operative leg, Strength_MVIC_ and Power_MVPO_, increased 107% and 93%, respectively. Knee flexion and extension range of motion of the operative leg improved following rehabilitation. 

## 4. Discussion

The aim of this preliminary study was to describe the use of an eccentrically-biased postoperative rehabilitation program targeted at deficits in physical function and muscle function in the early postoperative period following TKA surgery. Clearly, without a control or comparison group it is not possible to determine if the six-week (12 session) eccentrically-biased rehabilitation program induced the outcomes in this small sample of patients. Furthermore, any comparisons to existing literature should be made with caution since criteria for inclusion in previous studies are not identical and participant characteristics are likely different. The effects on the primary physical function endpoints, however, seem impressive. The normative levels of physical functioning, 50 on the SF-36_PCS_ [32], were reached and these outcomes were attained sooner (by the third postoperative month) than previously demonstrated [8, 9, 12, 13, 15, 45–62]. Further, the SF-36_PCS_ outcomes from our preliminary study were more than 10 points greater than those reported when outpatient rehabilitation was not utilized [8, 45–48, 50, 52–60] and more than 5 points greater than studies that utilized outpatient rehabilitation [12, 13, 15, 49, 63]. Our 6MW findings at three months also exceed the mean 6MW results one year following surgery in previous studies [9, 12, 15, 23, 64], and reached normative levels [12, 65, 66].

When the focus of postoperative rehabilitation is based on functional activities, short-term quality of life improvements are noted when compared to home exercise and education with small to moderate weighted means differences [22]. Studies that have utilized rehabilitation approaches focused on muscle conditioning also demonstrate improvements in physical functioning [12, 13, 15, 23], but only one randomized controlled trial [15] has compared progressive leg-strengthening exercises over six weeks to a standard of care group. While the 23 sessions of resistance exercise demonstrated superior strength and mobility improvements over the standard of care at one year, there were no group differences in physical function as depicted by the SF-36_PCS_. In our small descriptive study, the effect size (ES) of an eccentrically-biased rehabilitation program on physical and muscle function was large (ranging between ES = 1.5 and ES = 3.2). Every participant's SF-36_PCS_ change score and over one half of the 6MW change scores exceeded the minimal clinically important difference for those measures of physical function. Overall, the level of physical function at the third postoperative month following an eccentrically-biased rehabilitation program could be considered equivalent to or better than the levels achieved in the previous RCT at the one year. Moreover, the secondary physical function endpoints in our study, that included assessments of perceived (LEFS) and performance-based abilities (SCT and GS), demonstrated large effects (ranging from ES = 1.2 to ES = 1.7) and 54–100% of the participants' change scores exceeded clinically important levels. These improvements in the secondary endpoints are important since the LEFS better represents functional activities as compared to the WOMAC [67] and the SCT is one of the most demanding tasks for individuals after TKA [11].

The effect on strength over the first three postoperative months also appears greater with an eccentrically-biased program, though direct comparisons to earlier studies are challenging. Leg extension power (power_MVPO_), an important variable related to measures of physical function [41] following TKA [42, 43], also improved. The eccentrically-biased rehabilitation program induced large effects (ES = 1.5–1.9) in the strength and power of the operative leg. It is our contention, and our descriptive data supports this notion, that RENEW with its high force producing abilities can amplify the muscle conditioning and may mediate enhanced physical function [23]. Further, progressive strength loss of the nonoperative leg, thought to be predictive of outcomes following TKA [64], was not seen in our cohort. In this preliminary report, we demonstrate improvements in muscle function in both the operative and nonoperative legs which could be attributed to the bilateral resistance exercise of RENEW. 

This is the first report of the use of eccentrically-biased rehabilitation program for the knee and hip extensors early after TKA surgery. There were no adverse events associated with this rehabilitation approach. Eccentric exercise of the knee flexors has been tested previously as a rehabilitation intervention designed to reduce terminal knee extension deficits [68], but not as a strength-training mode of exercise early after TKA surgery. Our laboratory has specifically studied the effects of eccentric-biased resistance training of the knee extensors on mobility function in older adults with a variety of comorbid conditions. In all cases, we have reported significant improvements in quadriceps muscle strength and size, and in the hallmark activities underlying physical function (e.g., the ability to walk, negotiate stairs, and respond to balance challenges) [23–27]. RENEW is a potent resistance exercise that has reversed long-standing strength and mobility deficits following TKA [23] and has been responsible for sustained improvement 8 months after RENEW in other orthopaedic knee surgery patients [69, 70]. Importantly, the high compliance with RENEW following TKA is promising; however, the low number of individuals who actually agreed to begin the program tempers the enthusiasm surrounding compliance.

The limitations of a quasiexperimental design with a nonblinded assessor and the inability to assign the positive findings strictly to RENEW warrant caution when extrapolating these results. Our TKA recipients underwent a multifaceted program of which RENEW was only one component. The enrolled participants were also a select group with initial high levels of function. This precludes a direct comparison to other treatment studies that incorporate a broader range on participants with less restrictive inclusion/exclusion criteria. Additionally, there was no control group to help decipher whether the positive findings were simply a result of a natural return of muscle and physical function. It may be that this cohort of participants was bound for a good result independent of whether they engaged in formal outpatient rehabilitation. Also, since the participants were only engaged in outpatient rehabilitation for 3 months, this prevents an assessment of long-term implications of this rehabilitation mode. Since it has been demonstrated that the greatest rehabilitation effects on muscle conditioning occur during the first three months following TKA surgery, and that is the typical timing and duration of postoperative rehabilitation, this limitation, however, may be one of this study's greatest strengths. That is, the findings after 12 outpatient rehabilitation sessions over three months result in normal or near-normal physical function. While we are not able to comment on whether the muscle function changes were a result of improved activation or hypertrophy of the leg muscles, we do know the effect was large. In the future, we hope to perform a randomized, controlled study with a mediation analysis that will isolate the “active muscle ingredient” following TKA surgery that constitutes the causal pathway toward normalized physical function.

## 5. Conclusions

Utilizing RENEW as the strengthening mode of an eccentrically-biased rehabilitation program early after TKA contributed to changes in physical function to norm-based levels. This result alone suggests that the high muscle force production potential of eccentric exercise, at relatively low levels of exertion via RENEW, may contribute to an amplified level of physical function. The potential impact on best-practice rehabilitation following TKA is far reaching as greater focus on quadriceps strengthening is feasible and may be capable of optimizing outcomes. 

## Figures and Tables

**Table 1:** Participant demographics.

Subject	Sex	Age	BMI
A	F	59	23.8
B	F	53	21.0
C*	F	68	30.1
D*	F	54	34.0
E	F	52	54.8
F	M	43	26.4
G	M	51	23.1
H	M	65	43.3
I	M	62	28.4
J	M	54	26.5
K	M	60	36.0
L*	M	62	33.3
M	F	56	28.3

Mean		56.8	31.5
SD		6.7	9.2

*Staged bilateral (>6 months apart).

**Table 2:** The six-week outpatient rehabilitation protocol performed two times per week.

Outpatient rehabilitation protocol	
Warm up (15 min)	
Stationary cycling	5–10 min
Seated or supine AROM knee flexion and extension	2-3 min
Alternating ankle dorsiflexion and plantar flexion	1-2 min
Passive quadriceps stretching (standing or prone)	1-2 min
Passive hamstring stretching (standing or seated)	1-2 min

Leg strengthening exercise (30 min)	
RENEW 5–20 minutes (Table 3)	
Leg press 2 × 10–15 reps 70% 1 RM	
Leg extension 2 × 10–15 reps 70% 1 RM	
Leg curl 2 × 10–15 reps 70% 1 RM	
Standing calf raise 2 × 10–15 reps Body weight	

Functional task-oriented exercise (5 min)	
Get up and sit down	15 reps
Wall sits at 60 degrees 5–10 sec holds	15 reps
Negotiating stairs (stepups starting at 4′′ and progressed to 8′′)	30 steps
Body weighted half-squatting	15 rep
Unilateral standing firm and/or unstable surface (build up to 30 sec holds)	3–5 reps
Walking backward, forward, marching and side step on a slope, and/or with resistance	30 m

Endurance exercise (10 min)	
Treadmill walking Change of speed or on incline	5 min
Stationary biking “somewhat hard” effort	5 min

**Table 3:** Perceived exertion and resistance exercise (RENEW) progression (frequency and duration) over the 6-week training.

Training week	Time/week	Training duration	Rating of perceived exertion
1	2	5–8 minutes	7 (very very light)
2	2	11–14 minutes	9 (very light)
3	2	17–20 minutes	11 (fairly light)
4	2	20 minutes	11–13 (fairly light to some what hard)
5, 6	2	20 minutes	13 (somewhat hard)

**Table 4:** Physical and muscle function results: pre- to postrehabilitation changes, effect size and 95% confidence intervals.

		Prerehabilitation	Postrehabilitation	Δ Score	95% CI	Effect size
SF-36_pcs_ (out of 50)		31.9 ± 7.0	50.7 ± 4.3*	18.8	14.8, 22.8	3.2
Six-minute walk test (m)		354.6 ± 119.6	521.9 ± 106.3*	167.3	101.8, 232.8	1.5
LEFS (out of 80)		39.9 ± 16.5	61.9 ± 7.5*	22.0	11.7, 32.3	1.7
Knee extension deficit (deg)	Operated	10.7 ± 5.5	2.8 ± 3.2*	8.0	−5.3, 10.1	1.8
Knee flexion ROM (deg)	Operated	102.0 ± 8.1	113.8 ± 8.1*	11.8	−3.9, 16.8	1.5
Strength_MIVC_ (Nm)	Operated	52.9 ± 19.5	109.6 ± 36.6*	56.7	41.3, 72.1	1.9
	Nonoperated	146.3 ± 51.2	152.5 ± 53.2	6.2	−4.2, 16.5	0.1
Power_MVPO_ (W)	Operated	83.6 ± 39.2	161.0 ± 63.6*	77.4	53.4, 101.3	1.5
	Nonoperated	198.8 ± 104.0	222.2 ± 108.0*	23.4	11.1, 35.7	0.2
Stair climbing test (s)		17.2 ± 6.8	9.2 ± 3.9*	8.0	4.4, 11.6	1.4
Gait speed (m/s)		1.0 ± 0.3	1.3 ± 0.2*	0.3	0.2, 0.5	1.2

*Significant differences compared to pre-rehabilitation value (*α* < 0.01).

SF-36_PCS_: Physical component summary of the short form-36.

LEFS: Lower extremity function scale.

ROM: Range of motion.

Strength_MVIC_: Knee extension maximal voluntary isometric contraction.

Power_MVPO_: Leg extensor maximal voluntary power output.
